# Using New Instruments of Clustering Policy in the Health Care System. The Case of Poland

**DOI:** 10.3389/fphar.2016.00177

**Published:** 2016-06-21

**Authors:** Piotr Romaniuk, Tomasz Holecki, Joanna Woźniak-Holecka

**Affiliations:** ^1^Department of Health Policy, School of Public Health in Bytom, Medical University of Silesia in KatowiceBytom, Poland; ^2^Department of Health Economics and Health Management, School of Public Health in Bytom, Medical University of Silesia in KatowiceBytom, Poland; ^3^Department of Health Promotion, School of Public Health in Bytom, Medical University of Silesia in KatowiceZabrze, Poland

**Keywords:** economic clusters, health policy, health care organization, regioinal development, Poland

## Abstract

The issue of clusters as a form of organization of market entities has recently attracted an increasing attention of health care management theoreticians and practitioners. In our opinion the existing theoretical basis gives a foundation for considering clusters as a source of potential for increasing the effectiveness of health policy and health care organizations. It can be assumed that in case of health care clusters there is a possibility of interregional diffusion of innovation, based on ventures undertaken on the health care market, increasing not only the potential of the entities in the cluster, but also of its surroundings and subcontractors. It is possible to realize the idea of a flexible health care implemented regionally with the use of modern techniques of communication, knowledge transfer and high specialization. Nonetheless, in case of Poland the potential of clustrification remains untapped, being characterized by a limited actions of public and private bodies, marginal role of non-profit sector organizations and limited engagement of R&D sector. This is because a general distrust in the cluster formula, and the lack of relevant knowledge among local officials and health business leaders. For this reason the process of clustrification among health care entities requires external support through the increased efforts to create a system of legal and tax preferences for cluster initiatives and provision of organizational support in terms of know-how, targeted particularly at bodies and individuals, who may act as cluster leaders.

## Introduction

The issue of clusters as a specific form of organization in the area of economic policy, including health policy, has recently attracted an increasing attention of health care management theoreticians and practitioners. Although the phenomenon of clustering itself was observed as early as the end of the 19th Century (Feser and Luger, 2003), it was the last few decades, when the concepts on how to define a cluster, what are the cluster forms and methods of their scientific investigation, as well as what are the methods of creating and supporting them, started to develop very rapidly. Literature review leads to the conclusion that the functional branches for which the cluster concept has reference, are constantly expanding, covering more and more areas of social and economic life. Based on this trends it is also possible to consider, whether the development of clusters in the health care system could provide an organizational answer for the rising demand for medical services determined by the inevitable process of demographic change, which Poland and Europe are going to face in the next few decades.

Cluster applied on the health care market is quite a vague matter. The recent results of research on it have developed a theoretical material located in an interdisciplinary context, related to regional policy, entrepreneurship and health sciences. In our opinion this theoretical basis gives a sufficient foundation for considering clusters, along with the collaborative links being its feature, as a source of yet unused potential for increasing the effectiveness of health policy.

## What are clusters

One of the first cluster definitions was proposed by M.E. Porter, who defines it as “a geographical proximate group of interconnected companies and associated institutions in a particular field, linked by commonalities and externalities” (Porter, 2000). E. M. Bergman and E. J. Feser define cluster more narrowly as “close ties to certain companies and operators in terms of various aspects of their joint activity” (Bergman and Feser, 1999). These authors however also draw the possibility of defining cluster in a very broad way, as “a group of business enterprises and non-business organizations for whom membership within the group is an important element of each member firm's individual competitiveness” (Bergman and Feser, 1999).

The legal definitions of cluster differ depending on the actual source. In the Polish legislation this issue remains somewhat unclear, even though in the strategic documents clusters are perceived as a preferred form of economic cooperation (Rozporządzenie Ministra Gospodarki z dnia 2 grudnia, 2006; Rozporządzenie Ministra Rozwoju Regionalnego z dnia 7 kwietnia, 2008; Olko et al., 2011; Dzierżanowski, 2012)^1^. The European Commission documents in turn, instead of the cluster concept, used to apply a wider category of “innovative cooperation network” (Regional Clusters in Europe, 2002; W kierunku światowej klasy klastrów w Unii Europejskiej, 2008). In this case clusters are presented as groupings of independent businesses, emerging innovative companies, small, medium and large entities and research organizations that operate in a particular sector and in a particular territorial area. These structures are designed to stimulate innovative activity by promoting intensive contacts, sharing of facilities and exchange of knowledge and experience, in order to facilitate technology transfer, networking and dissemination of information within the organization (Stawicki, 2008).

The geographical concentration of entities in the cluster tend to appear mainly in the highly urbanized areas, where there is a natural tendency to cooperate, arising of the territorial proximity, as well as the density of entities being potential cluster members. Additionally, the specific location of the cluster, as well as the expected results of its emergence, makes the local authorities a natural partner participating in its activity, particularly at the regional level. In such a case, cluster becomes a kind of redefinition of the traditional approaches to the economic policy. The main participant of the competitive market game tends to change—from self-interest-oriented business entity, as assumed in the classic neo-liberal theories, to the regional community represented by its authorities. The role of the administration becomes to initiate and moderate the entire process of cluster creation and provide it with a specific support (Borras and Tsagdis, 2011). At the same time, clusters and the internal rules of these organizations are becoming similar to the concepts of interpersonal interactions and relationships, in social sciences commonly referred to as “social capital” (Vassileva, 2009).

The basic premise for clustering is the commonality of the benefits gained by all cluster participants, but natural beneficiaries are also members of the local community and the local authorities. They may gain purely economic profits, like increased budget revenues, or higher increase of employment. But such benefit may appear also as higher quality and availability of specific services or goods in a given region. In case of the business enterprises, the benefit is based primarily on ensuring the stability of demand for goods and services they offer on the regional market. They may also get better perspectives for market expansion, thereby increasing their competitive advantage outside of the region. Considerably important is also the reduction financial costs, social barriers and administrative procedures related to business activity. For other legal entities possibly participating in clusters (research institutions, NGOs) benefit will be primarily to provide favorable conditions from the point of view of their statutory objectives, and thus ensure the stability of the organization's existence (Stawicki, 2008; Borras and Tsagdis, 2011).

## Clusters in health care

In face of the characteristics of modern economic systems, regional policy (Madej, 1998; Mayer, 1999; Poniatowicz, 1999; Sługocki, 2004; Głowacki, 2005; Waldziński, 2005; Sokołowicz, 2008) can be treated as an interesting area of synthesis of issues concerning cluster-based policy and health policy. The role of the latter one is assuming that companies operating in the area of health, including units involved in provision of health services, and manufacturers of medical or pharmaceutical technologies, can be an important factor improving the regional economic competitiveness. Of significance is also the fact that medical industry is among the branches strongly contributing to the growth of innovative products and technologies available on the market. In other words, modern health care has to be seen as a developing branch of the national economy, which may be a basis for building development-oriented regional strategy with regard to its innovative features and competitive advantage. In light of the technological, demographic, political and economic changes the world is experiencing, further expansion should be expected, beyond the borders of the narrowly defined medicine serving the patient. Obvious is also the mutual relationship between health and economic growth. On the one hand, health is an important factor of economic development, on the other one—economic growth has a significant, positive impact on the health of the population, affecting the overall level of welfare. Beside of that, due to the compliance of purposes and aspirations, initiatives in the health care market can provide a platform of harmonious cooperation between public and private partners. In such circumstances, clusters seems to appear as a potentially very useful tool to be used also in this sector of activity.

One of the features of health care clusters is their broad spectrum of interests. Actually, any initiative, which may bring positive impact on the improvement or protection of health status of individuals or populations, may be qualified to this group. In case of absence of a clear strategic vision, this wideness might be an obstacle for the development of cluster. Nonetheless, in context of health objectives, such a wide margin of inclusion for potentially positively impacting initiatives, should be perceived as advantage. Businesses of almost any industry can find their own areas of action on the health care market. Scientific institutions can also find a space for developing innovations in an almost unrestricted manner. Finally, public administration is by law required to support the initiatives positively influencing public and individual health on their area of competence.

When understanding the concept of health as a public good, in connection with the metropolitan regionalism and entrepreneurship based on clustered collaboration, a previously mentioned role of social capital as a foundation for mutual projects based on trust, cooperation and constructive competition becomes a key factor (Brodzicki and Szultka, 2002; Sztompka, 2006; Romaniuk, 2011). In view of the concepts of regional policy and cluster-based policy, in connection with the features of health industry, it can be assumed that also in case of health care clusters there is a possibility of interregional diffusion of innovation, based on ventures undertaken on the health care market, increasing not only the potential of the entities in the cluster, but also of its surroundings and subcontractors. It is possible to realize the idea of a flexible health care implemented regionally, with the use of modern techniques of communication, knowledge transfer and high specialization.

These statements are confirmed by the experience of the existing health care clusters in Poland. According to G. Bigaj^2^, health care cluster is a platform of understanding and cooperation of all elements of the health care system. The result of its existence and activity should be the elimination of the health care system imperfections. Thanks to the implementation of new technologies, procedures, materials and medicines an improvement should be observed in the sphere of organization of services, service quality, and effectiveness of treatment. Among the objectives of such a cluster, there is a creation of networking between medical institutions which provide—on the principle of complementarity—medical services to an open group of patients. Another aim is also to support providers in relations with the public payer, administration, state's institutions and local self-government units. However, the principal aim of the cluster initiative is a partnership that will provide members with faster and more effective achievement their assumed objectives. Because the medical industry is one of the most cost-intensive, a well-organized cooperation of specialized entities means a greater chance of obtaining the necessary resources. E.g., Medical Cluster of Wielkopolska focuses on three areas: new medical technologies, improving management systems of health care centers, including the computerization of management processes and the functioning of the health system.

Despite such opinions and experiences, in Poland the potential of clustrification currently seems to be untapped. During our study of health care clusters in Poland we found moderate will for participation of public administration units and for-profit organizations, marginal role of non-profit sector organizations and limited engagement of R&D sector. Paradoxically, this is not because the health service is being included in a specific sub-market, which tends to be perceived as less profit oriented subject of public responsibility. As the studies have shown, local governments deciding to get involved in health care cluster initiatives, treat them in a manner analogous to structures operating in other market sectors. At the same time, there is observable higher involvement in such initiatives by the units identifying on their territory the existence of a key medical product (Holecki and Romaniuk, 2016). This observation suggests that local governments start to recognize the potential lying in the use of the cluster formula within the health services market. This also confirms the features of health industry, as an important contributor to the local economic system and its competitiveness, moreover that decision-makers, business collaborators, providers, and sometimes even patients, are willing to consider it in such a way. Thus, medical services can strengthen the competitive advantage of Polish regions, but should be preceded by a thorough diagnosis of the initial state, which in this specific market requires particular precision, political and economic balance of arguments and social consensus.

The limited use of cluster formulas is reflected in the small-scale support to clusters, as well as in the nature of this support, largely limited to declarations only, not a real operations. The reason for this seems to be a general reluctance of possible cluster members to use this formula, arising of distrust, but also lack of relevant knowledge among local officials and health business leaders, disabling them to engage in the development of clusters, even if there is a will to do so.

In Poland the number of health care clusters, even if to apply its broad definition, including organizations active in medicine, pharmacy, medical products supply and public health initiatives, is limited and concentrated in the traditional sectors. The situation is different in West European countries, where the development of medical sciences makes the business areas for health care clusters to constantly evolve and expand the scope of their activity to new areas, such as e-health and m-health. They are using new sub-markets and technologies to stimulate their own economic success, but also to strengthen the economy of the region or country, while ensuring their citizens access to the latest technological achievements. Depending on the specialization, they can associate enterprises from various industries, both small and medium-sized, just entering the market, or having longer experience. Some clusters are composed of hundreds of companies, others are limited to a few or several entities. In larger countries, such as Germany or France, they are concentrating mainly on regional dimension, often being stimulated by renowned academic centers. There are first examples associating companies from several countries, such as the Brussels Life-Tech Cluster, which consists of over 600 companies from 14 countries. There are also first examples of cooperation between clusters themselves (Kaczmarek and Iltchev, 2015).

Worth noting are the cases of implementation of the clustering model based on the WHO's strategy “Health 2020.” An example of this might be a primary health care reform project applied in Hungary, which was referring to the concept of clusters as network of cooperating providers, who tend to expand the catalog of classical health services with a wide spectrum of activities in the area of disease prevention and health promotion (Adany et al., 2013; Jakab, 2013). Among the examples of health care clusters in Poland, there are interesting cases of groupings aimed at development of health services provided internationally, thus providing attempt to implement solutions to increase the competitiveness of Polish health care providers, under the provisions of the EU directive on cross-border health care^3^^,^^4^.

## Conclusions

The expansion of cluster initiatives stimulated by the economic law, which can be observed in many sectors of modern economies, most probably will appear also in the health sector. In light of recent clustrification experience in this sector, as well as opinions and expectations expressed by health market participants and experts specializing in investigating this sector, it remains an open question, what final form this process is going to take. At least two possible directions of evolution of these organizational solutions should be expected. On the one hand, due to the strong fusion between health care market and public administration, which in the vast majority of developed countries is embarked with the responsibility for ensuring access to health care services to the population, we can expect clustering to become a tool used for increasing the efficiency of this process. On the other hand, it may become just a tool of commercialization of ideas, taking a form analogous to, or only slightly differing from, clusters already existing in other markets. It is worth noting that in the light of the considerations outlined in this article, both of these forms can develop with a strong stimulation on the side of the state's economic and social policy. In both cases, however, slightly different will be the objectives of such stimulation. In the first case the assumed beneficiary of clustrification will be the community living in a given geographical area, and the expected effect of government's stimulus measures will be to improve the efficiency of health services, reduce their costs and increase availability, so in terms of general application—to increase the effectiveness of the impact on public health. Although in this case, similarly to the second one, we have to deal with the combination of commercial and non-commercial entities, bodies directly involved in providing health services, and those engaged rather in development and delivery of new technologies, units from the area of economy, science or social organizations—their advantage will be rather a secondary to the profit generated in the sphere of health system and social life.

In the second case the assumption of public stimulation will be primarily to support the competitiveness of companies operating in the health care market. This may apply to service providers and to companies specializing in developing modern medical technologies, pharmaceuticals, or the broadly recognized know-how. The average receiver of health care benefits also in this case can gain some profit, like the access to services at a higher level of technological advancement, with potentially better therapeutic effectiveness and quality. It will be, however, more a side effect of stimulus measures addressed primarily to the market participants, as well as supporting the economic development of regions, where health care will become one of the elements contributing to the competitiveness of geographically concentrated economic system.

The previous experience with clustrification in health care suggest dominance of the second of described trends. Moreover, in a relatively small scale the observable processes are actually the result of intentional supportive actions taken by the public administration. To a greater extent they are a result of bottom-up initiatives taken by the stakeholders. At the same time health care organizations emphasize the great potential inherent in the clustrification process, while among the obstacles to its dynamization, they indicate mainly the deficit of trust and lack of leaders who would be willing to take on the burden of initiation and ongoing coordination of the development and functioning of the cluster. It is also commonly underlined that the large scope of potential benefits of clustrification may be obtained more in the social sphere, than on the market. In our opinion, in the area of public health policy there is the space for providing the social sphere and the market with the expected coordinative solutions. At the same time it is possible to manage the affected processes in such a way, as to maximize the chances of achieving the desired positive economic effects, and optimizing internal processes taking place within the health system. Thus, in our opinion, health care cluster can be an innovative tool for the harmonization of public interest and the particular interests of a purely economic nature, that in the public debates on health are often situated in an antagonistic position to each other.

The example of Poland proves that the process of clustrification among health care entities requires external support, which should be firstly understood as the activity of public institutions. This is all the more justified, that the formula of public-private symbiosis, organizational and financial co-responsibility, is a favorable solution from the point of view of the expected health outcomes and efficiency. Despite this fact, the case of Polish public administration is that it do not provide enough support for entities willing to take action in health care with the use of economic cluster. Therefore, it appears advisable to formulate a package of recommendations for state's policy to support clustrification of health care through the increased efforts to create a system of legal and tax preferences for cluster initiatives and provision of organizational support in terms of know-how. This support should be targeted particularly at bodies and individuals, who may act as cluster leaders.

## Author contributions

PR and TH delivered the data for the study and outlined the paper's key messages. JW prepared the draft of the paper. TH contributed to the preparation of the final version of the paper and provided new information necessary to revise the paper. PR prepared the final version of the paper.

### Conflict of interest statement

The authors declare that the research was conducted in the absence of any commercial or financial relationships that could be construed as a potential conflict of interest. The reviewer SK and handling Editor declared their shared affiliation, and the handling Editor states that the process nevertheless met the standards of a fair and objective review.

## Footnotes

^1^www.mg.gov.pl (accessed 17 April, 2016)

^2^A leader of the Medical Cluster of Wielkopolska—personal interview.

^3^www.pikmed.pl/centrum-informacji-ds-transgranicznej-opieki-zdrowotnej (accessed 10 May, 2016)

^4^http://medycyna.lublin.eu/o-klastrze/wizja-misja-cele/?lang=en (accessed 10 May 2016)
